# Immunosuppressive effects of a novel potassium channel toxin Ktx-Sp2 from Scorpiops Pocoki

**DOI:** 10.1186/s13578-019-0364-1

**Published:** 2019-12-16

**Authors:** Yubiao Zhang, Feng Zhang, Shujuan Shi, Xinqiao Liu, Weisong Cai, Guangtao Han, Caihua Ke, Siru Long, Zhiyong Di, Shijin Yin, Haohuan Li

**Affiliations:** 1 Department of Orthopedics, Renmin Hospital of Wuhan University, Wuhan, 430060 People’s Republic of China; 2 School of Pharmaceutical Sciences, South-Central University for Nationalities, Wuhan, 430074 People’s Republic of China; 3 School of Life Sciences, University of Science and Technology of China, Hefei, 230027 People’s Republic of China

**Keywords:** Scorpiops Pocoki, Kv1.3 channel, Genetic engineering, Immunosuppression, IL-2

## Abstract

**Background:**

The cDNA Library of venomous animals could provide abundant bioactive peptides coding information and is an important resource for screening bioactive peptides that target and regulate disease-related ion channels. To further explore the potential medicinal usage of the transcriptome database of Scorpiops Pocoki’s venom gland, this research identified the function of a new potassium channel toxin Ktx-Sp2, whose gene was screened from the database by sequence alignment.

**Results:**

The mature peptide of Ktx-Sp2 was obtained by genetic engineering. Whole-cell patch-clamp experiment showed that Ktx-Sp2 peptide could effectively block three types of exogenous voltage-gated potassium channels—Kv1.1, Kv1.2 and Kv1.3, among which, the blocking activity for Kv1.3 was relatively high, showing selectivity to some extent. Taking Jurkat T cells as the cell model, this study found that Ktx-Sp2 peptide could also effectively block endogenous Kv1.3, significantly reduce the free calcium concentration in Jurkat T cells, inhibit the activation of Jurkat T cells and reduce the release of inflammatory cytokines IL-2, showing a strong immunosuppressant effect.

**Conclusions:**

This study further proves that the transcriptome database of the Scorpiops Pocoki venom gland is an important resource for discovery of novel bioactive polypeptide coding genes. The newly screened Kv1.3 channel blocker Ktx-Sp2 expanded the range of leading compounds for the treatment of autoimmune diseases and promoted the development and application of scorpion toxin peptides in the field of biomedicine.

## Introduction

Scorpion is one of the oldest species on earth. It has a great diversity and distributes widely in major terrestrial ecosystems. According to incomplete statistics, there are about 20 families, 208 genera and 2231 species of scorpion [1]. When hunting, a pair of venom glands in the tail of a scorpion release neurotoxic venom to paralyze or kill its prey. The venom is extremely important for the survival of the scorpion. Scorpion venom has complex components, including protein and non-protein components. The non-protein components mainly contain mucopolysaccharides, lipids, inorganic salts, nucleotides, free amino acids and biological amines. In contrast, the protein part is more abundant, not only including certain amount of phosphatase, hyaluronidase, metalloproteinase and other enzymes, but also bioactive peptides which selectively act on many kinds of ion channels [2]. With the increasing abundance of high-throughput screening technologies, researches on the composition, structure and function of scorpion venom are also deepening, and many of scorpion venoms have become the leading substances for the development of new drugs or important molecular tools for pathophysiological research [3].

Tibet is located in the southwest border of China. It has a vast territory and a special geographical environment, which provides a ideal habitat for Tibetan scorpion species, and leads to the evolution of endemic Tibetan scorpion species, including the *Scorpiops Pocoki* [4]. Transcriptome study has shown that *Scorpiops Pocoki* venom gland is rich in bioactive polypeptide encoding genes targeting certain ion channels, indicating its potential medicinal application [5]. In this study, a new toxin peptide encoding gene *Ktx*-*Sp2* was found from the transcriptome database of the *Scorpiops tibetanus* venom gland. Its encoded mature peptide is highly similar to α-potassium channel toxin peptides LmKTx10 [6] and II.10.5 [7], suggesting that it might be a new Kv1.3 channel blocker and have immunomodulatory effects. Due to the fact that *Scorpiops Pocoki* has not been domesticated in Tibet, it cannot be bred on a large scale, and it is not possible to directly extract mature Ktx-Sp2 peptide from the *Scorpiops Pocoki* venom. This study uses prokaryotic expression purification technology to prepare Ktx-Sp2 by genetic engineering, and tests whether it can block the Kv1.3 channel and produce immunosuppressive effect with electrophysiology recording, calcium imaging and immunology technologies.

## Materials and methods

### Construction of expression vector pGEX-4T-1-*Ktx*-*Sp2*

**Fig. 1:**
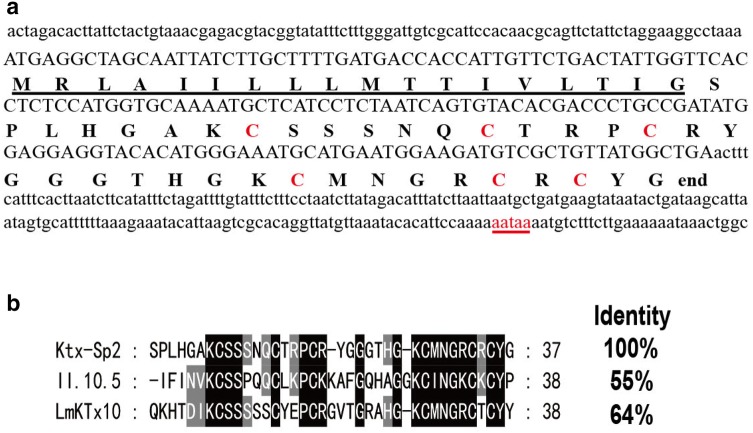
**a** Full-length nucleotide and the corresponding amino acids sequences of *Ktx*-*Sp2*. The signal peptide is underlined, while the potential polyadenylation signal aataa is underlined with red colors. Capital C in red color indicate the cysteine residues, 5′ and 3′ UTR regions are in lowercase letters. **b** Sequence alignments of peptide Ktx-Sp2 with the nearest neighbors II.10.5 and LmKTx10

Expression plasmid pGEX-4T-1-*Ktx*-*Sp2* was constructed on the basis of the full-length cDNA of *Ktx*-*Sp2* (Fig. 1a). Primers were designed to match the mature region of *Ktx*-*Sp2*. A second PCR used the products of the overlapping PCR as templates. The primers used were: Sense primer 1, 5′-CTGGGATCCGATGACGATGACAAGTCACCGCTGCATGGTGCAAAATGT-3′ with a BamHI restriction enzyme site (underlined) and corresponding to five codons encoding an enterokinase cleavage site (underlined twice); Sense primer 2, 5′-CATGGTGCAAAATGCTCATCCTCTAATCAGTGTACCCGTCCGTGCCGT-3′; Antisense primer 1, 5′-CCGCTCGAGTCAGCCATAACAGCGACAACGACCATTCATGCATTTACC-3′; Antisense primer 2, 5′-ATTCATGCATTTACCATGGGTACCACCTCCATAACGGCACGGA CGGGT-3′ with XhoI restriction enzyme site (underlined). The PCR products were inserted into expression vector pGEX-4T-1.

### Expression and purification of Ktx-Sp2 peptides

*Escherichia coli* Rosetta (DE3) cells containing pGEX-4T-1-*Ktx*-*Sp2* were proliferated at 37 °C in LB with 100 mg/ml ampicillin. Fusion protein synthesis was induced by the addition of 1 mM isopropyl β-d-thiogalactoside (IPTG) at 28 °C for 4 h. Cells were harvested and resuspended in glutathione (GSH) wash buffer (pH 8.0, 50 mM Tris–HCl, 20 mM EDTA), digested by 1 mg/ml lysozyme for 30 min. After a brief sonication, the extract was clarified by a centrifugation at 10,000×*g* for 15 min. The fusion protein was purified by GSH affinity chromatography and enriched by centrifugal filter devices (Millipore, 10 kDa). High performance liquid chromatography (HPLC) was used to further purify peptide, under the 230 nm wavelength to monitor the absorbance of the eluate at room temperature (22–25 °C). After cleavage of the fusion protein by enterokinase (More Biotechnology, Wuhan) for 8 h at 37 °C, the mixture was filtered (Millex-HV, 0.45 mm, Millipore) and separated on a C18 column (Elite HPLC, China, 10 mm × 250 mm, 5 μm) using a linear gradient from 10 to 80% CH3CN with 0.1% TFA in 60 min with a constant flow rate of 5 ml/min. Peaks were collected manually.

### Cell isolation, culture and ion channels expression

Mouse spinal columns were removed and placed in ice cold HBSS, then laminectomies were performed and bilateral DRG were dissected out. After removal of connective tissues, DRG were transferred to a 1 ml Ca^2+^/Mg^2+^-free HBSS containing 2 μl saturated NaHCO3, 0.35 mg l-cysteine and 20 U papain (Worthington, Lakewood, NJ, USA), and incubated at 37 °C for 10 min. The suspension of DRG was centrifuged, the supernatant was removed, 1 ml Ca^2+^/Mg^2+^-free HBSS containing 4 mg collagenase type II and 1.25 mg dispase type II (Worthington) was added and incubated at 37 °C for 10 min. After digestion, neurons were pelleted, suspended in neurobasal medium containing 2% B-27 supplement, 1% l-glutamine, 100 U/ml penicillin plus 100 μg/ml streptomycin, and 50 ng/ml nerve growth factor, plated on a 12 mm coverslip coated with poly-l-lysine (10 μg/ml) and cultured under a humidified atmosphere of 5% CO2/95% air at 37 °C for 18–24 h before use. Jurkat E6-1 T cells (ATCC TIB152) and HEK293T cells (ATCC ACS4500) were maintained in RPMI medium 1640 (Invitrogen, Carlsbad, CA, USA) and Dulbecco’s modified Eagle’s medium (DMEM) (Life Technologies,GrandIsland, NY, USA), supplemented with 10% fetal bovine serum (Life Technologies), 100 units/ml penicillin, 100 μg/ml streptomycin, respectively. Cells were cultured in a humidified incubator at 37 °C with 5% CO_2_. The cDNAs encoding mKv1.1, mKv1.2, mKv1.3, mNav1.4, mNav1.5 and mNav1.7 were subcloned into the XhoI/BamHI sites of a bicistronic vector, pIRES2-EGFP (Clontech, USA), then transiently transfected into HEK293-T cells using Lipofectamine 2000 (Invitrogen) for electrophysiological experiments.

### Whole-cell patch-clamp recordings

Whole-cell patch-clamp recordings were performed using an EPC 9 amplifier (HEKA Elektronik, Lambrecht/Pfalz, Germany) at room temperature (22–24 °C). Pipettes pulled from borosilicate glass (BF 150-86-10; Sutter Instrument Company, Novato, CA, USA) had resistances of 2–4 MΩ when filled with the internal solution. The internal pipette solution for recording Kv currents contained: KCl 140 mM, MgCl_2_ 1 mM, EGTA 1 mM, Na_2_ATP 3 mM, HEPES 10 mM (pH 7.3 with KOH); for recording Na currents contained: CsCl 100 mM, CsF 40 mM, MgCl_2_ 2 mM, HEPES 10 mM, EGTA 10 mM, d-Glucose 10 mM, Na_2_ATP 3 mM (pH 7.3 with CsOH). The external solution for recording Kv currents contained: KCl 5 mM, NaCl 140 mM, HEPES 10 mM, CaCl_2_ 2 mM, MgCl_2_ 1 mM, d-Glucose 10 mM (pH 7.4 with NaOH); for recording Na currents contained: NaCl 132 mM, KCl 5.4 mM, CaCl_2_ 1.8 mM, MgCl_2_ 0.8 mM, HEPES 10 mM, d-Glucose 5 mM (pH 7.4 with NaOH). Kv currents were elicited by + 50 mV, 400 ms depolarizing pulse from the holding potential of − 60 mV every 20 s, and Na currents were elicited by + 10 mV, 100 ms depolarizing pulse from the holding potential of − 60 mV every 10 s. Using IGOR (WaveMetrics, Lake Oswego, OR) software, concentration–response relationships were fitted according to modified Hill equation: I_toxin_/I_control_ = 1/1 + ([peptide]/IC_50_), where I is the steady-state current and [peptide] is the concentration of toxin. The parameter to be fitted was concentration of half-maximal effect (IC_50_).

### Live cell Ca^2+^ imaging

Jurkat T cells were loaded with 4 μM Fura-2 AM (Life Technologies) for 60 min at 37 °C. Cells were then washed 3 times and incubated in Hank’s Balanced Salt Solution (HBSS) for 30 min at room temperature before use. Fluorescence at 340 nm and 380 nm excitation wavelengths was recorded on an inverted Nikon Ti-E microscope equipped with 340, 360 and 380 nm excitation filter wheels using NIS-Elements imaging software (Nikon Instruments Inc., Melville, NY, USA). Fura-2 ratios (F340/F380) reflect changes in [Ca^2+^]_i_ upon stimulation. Data were obtained from 100 to 250 cells in time-lapse images from each coverslip.

### IL-2 secretion measurements

IL-2 secretion from Jurkat T cells was measured using an ELISA kit (eBioscience, San Diego, CA, USA) following manufacturer’s instructions. Cells were centrifuged at 1500 rpm for 10 min, and the supernatants were collected to measure IL-2 concentrations. Reactions were performed in 96-well plates coated with the capture antibody and stopped with phosphoric acid (1 M). Absorbance was measured at 450 nm. Each experiment was repeated at least three times in duplicate.

### Statistical analysis

All data are presented as mean ± SEM for n independent observations. Statistical analysis of differences between groups was carried out using paired t-test or ANOVA. P < 0.05 was considered significantly different.

## Results

### Sequence analysis of *Ktx*-*Sp2*

Through transcriptome analysis of Scorpiops Pococki venom glands, one of the nucleotide sequences obtained was selected. Its ORF encodes a new putative neurotoxin which was termed *Ktx*-*Sp2*. The precursor nucleotide sequence of *Ktx*-*Sp2* is 424 bp in length, including three parts: 5′UTR, ORF and 3′UTR. The 5′ and 3′ UTRs of *Ktx*-*Sp2* are 52 and 204 bp (Fig. 1a), respectively. At 3′UTR of the cDNA, a single AATAAA polyadenylation signal is found 28 nt upstream of the poly(A) tail. The 168 bp ORF encodes a precursor consisted of 56 amino acid residues (Fig. 1a). SignalP V4.1 server (http://www.cbs.dtu.dk/services/SignalP/) predicted that the precursor of Ktx-Sp2 contains a putative signal peptide of 18 residues followed by a mature toxin of 37 residues with three pairs of disulfide bridges. By sequence alignment with the other toxins (Fig. 1b), it is reasonable to assume that Ktx-Sp2 adopts the well-known cysteine-stabilized α/β scaffold, and is similar to the classical scorpion K + -channel blockers. The 64% and 55% identity of Ktx-Sp2 with II.10.5 [7] and LmKTx10 [6], two kinds of Kv1.3 channel blockers showed that Ktx-Sp2 may have the similar function of blocking Kv1.3 channels, yet it is necessary to investigate biological effects of Ktx-Sp2 peptide by electrophysiological experiments and identify its specific target.

### Expression, purification and identification of Ktx-Sp2 peptide

**Fig. 2:**
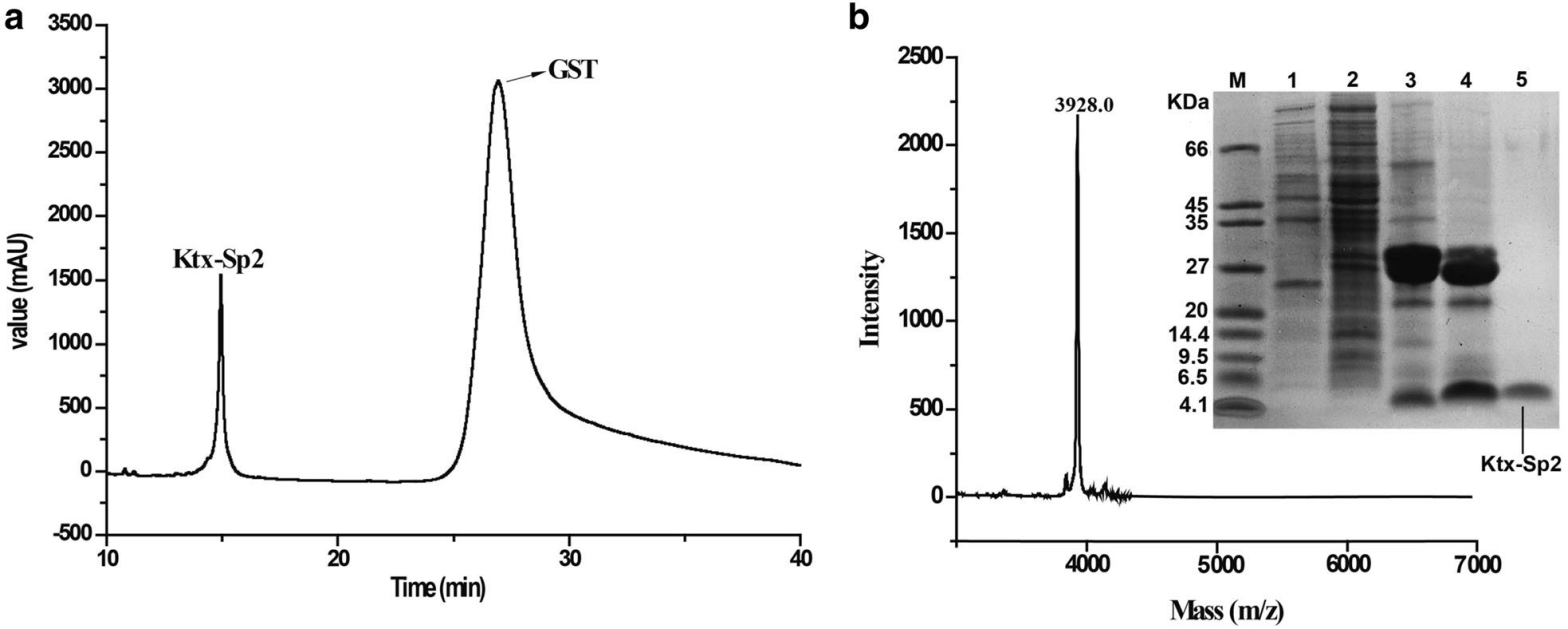
The expression, purification and identification of peptide Ktx-Sp2. **a** Purification of Ktx-Sp2 by HPLC on a C18 column. **b** Mass spectrum of Ktx-Sp2 peptide measured by MALDI-TOF–MS. Measured value is 3928.0 Da, and the calculated one is 3928.0 Da. Right inset shows the tricine/SDS-PAGE analysis of the purification of Ktx-Sp2 peptide. M, molecular mass markers; Lane 1, proteins from non-induced *E. coli* Rosetta (DE3) cells; lane 2, proteins from induced *E. coli* Rosetta (DE3) cell containing pGEX-4T-1-Ktx-Sp2 by IPTG; lane 3, spurified GST fusion protein after affinity chromatography and desalting; lane 4, fusion protein cleaved by enterokinase; lane 5, purified Ktx-Sp2 by reversed phase HPLC

The expressed GST-Ktx-Sp2 fusion protein was purified on GSH affinity column and then the purified GST-Ktx-Sp2 fusion protein was desalted using centrifugal filter devices. The fusion protein was cleaved by enterokinase to separate GST protein with Ktx-Sp2 peptides. As shown in Fig. 2a, the fusion protein of 30 kDa size was purified successfully and split into two products, the 26 kD GST and 4.1 kDa target peptide. The mixture were further separated by HPLC, which resulted in two peaks. The component eluting at about 14 min and corresponding to Ktx-Sp2 was collected manually and lyophilized. The molecular weight of Ktx-Sp2 was determined by matrixassisted-laser-desorption/ionization time-of-flight mass spectrometry (MALDI-TOF–MS). MALDI-TOF–MS showed the measured value of Ktx-Sp2 is 3928.0 Da (Fig. 2b) which is consist with the theoretical molecular weight of 3928.0 Da.

### Selective blocking of Ktx-Sp2 on exogenous Kv1.3 channel

**Fig. 3:**
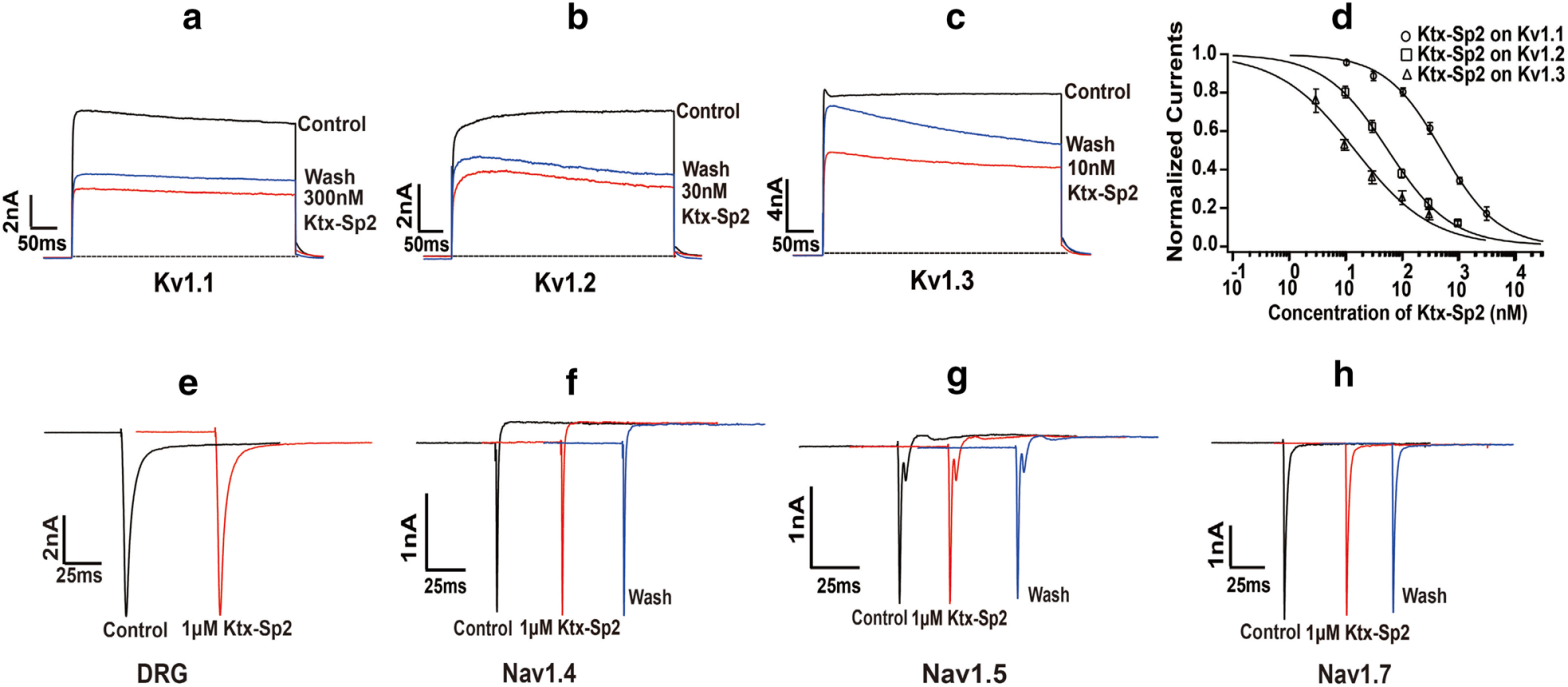
Selective blocking of Ktx-Sp2 on exogenous Kv1.3 channel. **a**–**c** Showed the current traces in the absence (Control) or presence of 300 nM, 30 nM and 10 nM Ktx-Sp2 on Kv1.1, Kv1.2 and Kv1.3 channels respectively. **d** Dose–effect curves of Ktx-Sp2 on Kv1.1, Kv1.2 and Kv1.3 channels. **e**–**h** showed the current traces in the absence (Control) or presence of 1 μM Ktx-Sp2 on endogenous sodium channels expressed by DRG and exogenous Nav1.4, Nav1.5, Nav1.7 channels heterologously expressed in HEK293T cells respectively

Sequence alignments showed that Ktx-Sp2 polypeptide has high homology with Kv1.3 channel blockers LmKTx10 and II10.5 (Fig. 1b), which suggested that Ktx-Sp2 may also have the function of blocking Kv1.3 channels. We first examined whether Ktx-Sp2 could block exogenous Kv1.3 channel expressed by HEK293T cells. As expected, Ktx-Sp2 reduced the peak amplitude of wild-type mKv1.3-mediated currents in a concentration-dependent manner with an IC_50_ of 14.72 ± 1.98 nM (n = 8) (Fig. 3c and d). Mammalian Kv1.1 and Kv1.2 are highly homologous to the Kv1.3 channel and affect the selectivity of Kv1.3 channel blockers, so we also observed the regulation of Ktx-Sp2 peptides on Kv1.1 and Kv1.2 channels heterologously expressed in HEK293T cells. Electrophysiological results showed Ktx-Sp2 exhibited about 4 and 33-fold selectivity for Kv1.3 over Kv1.1 (IC_50_, 484.99 ± 25.5 nM) (Fig. 3a and d) and Kv1.2 (IC_50_, 56.896 ± 2.32 nM) (Fig. 3b and d) respectively. To further investigate the selectivity of Ktx-Sp2 on Kv1.3 channel, we also tested the effect of Ktx-Sp2 on sodium channels. The addition of 1 μM Ktx-Sp2 almost had no effect on endogenous sodium channels expressed by DRG neurons (Fig. 3e) and exogenous Nav1.4 (Fig. 3f), Nav1.5 (Fig. 3g), Nav1.7 (Fig. 3h) channels heterologously expressed in HEK293T cells. These electrophysiological results suggested that Ktx-Sp2 could serve as a potential drug lead for selectively targeting Kv1.3 channel and would be very helpful in drug design for treating autoimmune diseases.

### Ktx-Sp2 inhibit endogenous Kv1.3 in Jurkat T cells

**Fig. 4:**
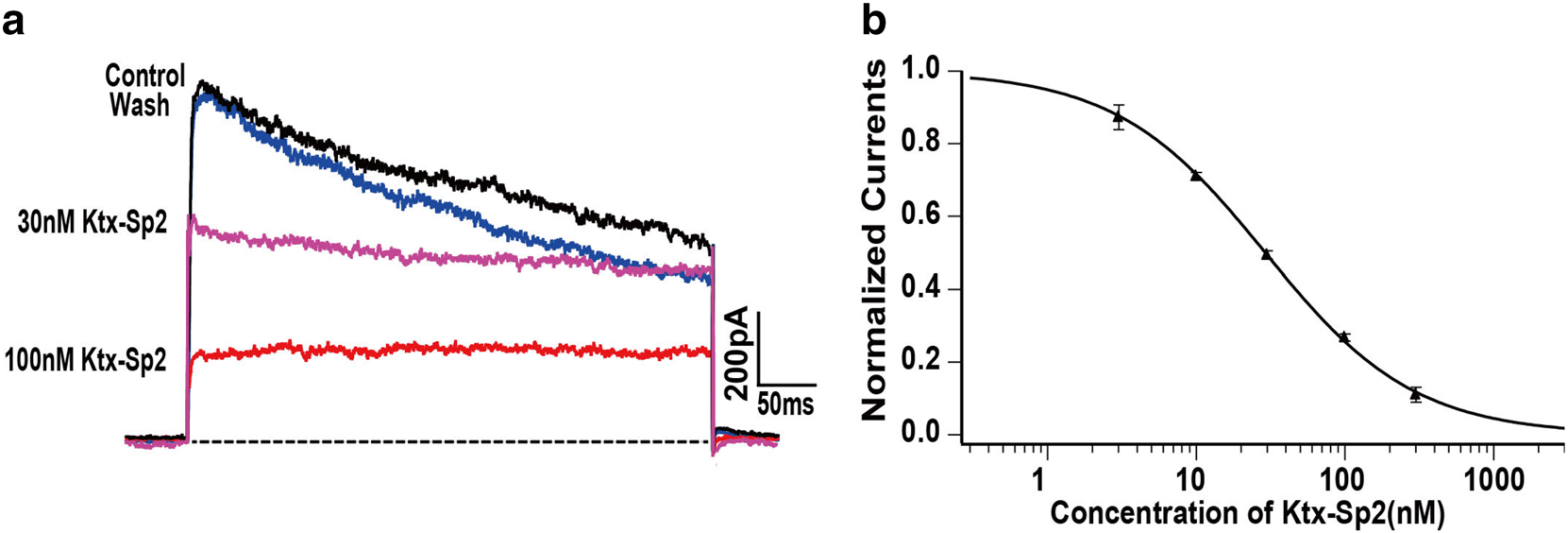
Ktx-Sp2 inhibit Kv1.3 endogenously expressed by Jurkat T cells. **a** Representative traces illustrate that Ktx-Sp2 inhibited the Kv1.3 current in a Jurkat T cell in a concentration-dependent manner. **b** Concentration–response curve of Ktx-Sp2 inhibition of Kv1.3 current in Jurkat T cells. Currents were normalized to the control and fitted by a Hill equation

Two major types of K^+^ channels are expressed by the human Jurkat T cells: Kv1.3 [8], and the small conductance Ca^2+^-dependent K^+^ channel (SKCa2) which is activated by a cytosolic Ca^2+^ rise [9]. We then examined whether Ktx-Sp2 could also regulate the activation of endogenous Kv1.3 expressed by human Jurkat T cells which is commonly used to study T cell signaling [10]. To avoid activation of the SKCa2 channel we used a pipette solution containing almost zero cytosolic Ca^2+^. Kv1.3-mediated currents were elicited by 400 ms depolarizing pulses to + 50 mV, from a holding potential of − 60 mV. Bath application of Ktx-Sp2 reduced Kv1.3 current by about 50% even at a low concentration of 30 nM (n = 5). The suppressive effect of Ktx-Sp2 was partially reversible after washout (Fig. 4a). The inhibitory effect of Ktx-Sp2 was concentration-dependent with the IC_50_ of 29.09 ± 1.12 nM (Fig. 4b).

### Ktx-Sp2 suppresses Ca^2+^ signaling in Jurkat T cells

**Fig. 5:**
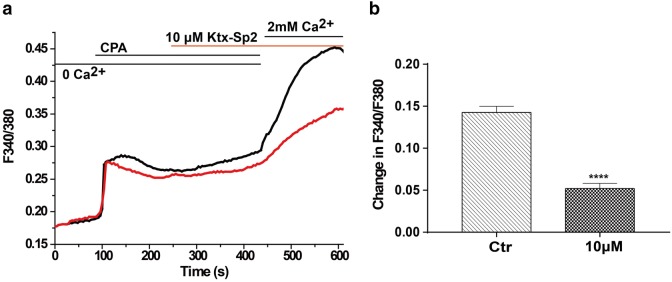
Inhibition of Ca^2+^ influx by Ktx-Sp2 in Jurkat T cells. **a** Representative traces show the effect of Ktx-Sp2 on store depletion-induced Ca^2+^ influx. Each trace represents averaged F340/F380 ratio from about 300 Jurkat T cells. The ratio traces in the presence of 0 and 10 μM Ktx-Sp2 are color-coded with black and red respectively. The first [Ca^2+^]_i_ peak presents a rapid Ca^2+^ rise evoked by 10 μM CPA in 0 Ca^2+^ in extracellular solution, and the second [Ca^2+^]_i_ peak illustrates the sustained store depletion-induced Ca^2+^ influx with an addition of 2 mM Ca^2+^ in the absence and presence of Ktx-Sp2. **b** Statistical data summarizes the net changes in F340/F380 ratios induced by 2 mM extracellular Ca^2+^, ****P ≤ 0.0001

As Kv1.3 is involved in generating the resting membrane potential which drives Ca^2+^ influx and contributes to Ca^2+^ homeostasis in T cells, and a previous study showed that inhibition of Kv1.3 reduces Ca^2+^ influx in Jurkat T cells [11], suggesting that Ktx-Sp2 should also reduce Ca^2+^ influx in Jurkat T cells. To test this hypothesis we performed live cell Ca^2+^ imaging using the Ca^2+^ indicator dye Fura-2 to determine the effect of Ktx-Sp2 on [Ca^2+^]_i_ in the presence of intracellular Ca^2+^ store depletion by cyclopiazonic acid (CPA), an inhibitor of the sarcoplasmic reticulum Ca^2+^-ATPase [12]. In the absence of extracellular Ca^2+^, 10 μM CPA induced a relative small, transient rise of [Ca^2+^]_i_ in Jurkat T cells. When 2 mM Ca^2+^ was added to the Ca^2+^-free HBSS, we observed a large Ca^2+^ influx into the cells. Pre-treatment of Jurkat T cells with 10 μM Ktx-Sp2 for 3 min produced a obvious inhibition of Ca^2+^ influx (Fig. 5a). At 10 μM, Ktx-Sp2 decreased the F340/F380 ratio from 0.14 ± 0.02 to 0.05 ± 0.01 (n = 278) (Fig. 5b). These results suggest that Ktx-Sp2 is a potent inhibitor of intracellular Ca^2+^ signaling of T cells through inhibition of Kv1.3.

### Ktx-Sp2 inhibits IL-2 secretion from activated Jurkat T cells

**Fig. 6:**
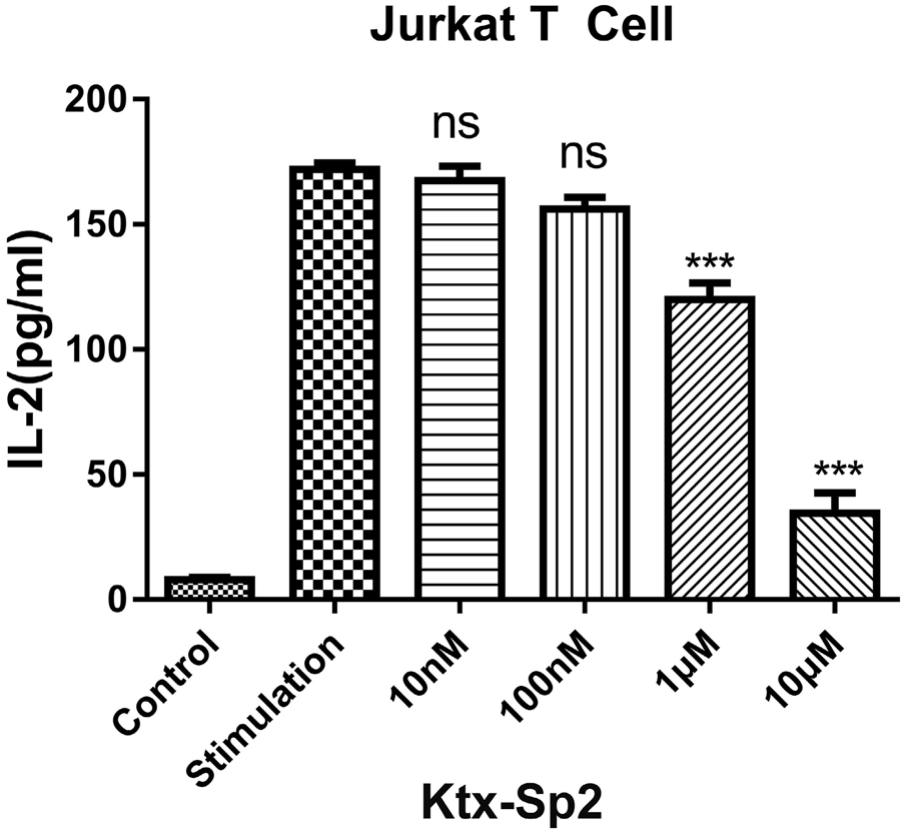
Ktx-Sp2 inhibits IL-2 secretion from activated Jurkat T cells. The concentrations of IL-2 in cell growth media were measured by ELISA. Jurkat T cells were activated by ConA (10 μg/ml) for 24 h. Ktx-Sp2 (10 nM, 100 nM, 1 μM and 10 μM) were added simultaneously with ConA. ***P < 0.0001, n = 6

The cytokine IL-2 is predominantly secreted from activated T cells and is critical in regulating the balance between immune tolerance and autoimmunity [13]. The secretion of IL-2 is driven by a rise of [Ca^2+^]_i_ [14]. Since Ktx-Sp2 potently suppresses Kv1.3 function and decreases Ca^2+^ influx in Jurkat T cells we next asked whether Ktx-Sp2 could inhibit IL-2 secretion, which should result in functional immunosuppression. As a classical polyclonal stimulator acting through T-cell receptor complex (TCR)-CD3 complex, ConA has been used to induce T cell activation and stimulate IL-2 secretion, which is dependent on an increase of [Ca^2+^]_i_ [15]. We thus used the ConA-stimulated IL-2 secretion in the growth media as readout. As predicted, IL-2 concentration measured by ELISA was increased markedly upon stimulation with 10 μg/ml ConA but not vehicle control (Fig. 6). Pre-treatment of the cells with different concentrations of Ktx-Sp2(10 nM, 100 nM, 1 μM and 10 μM)for 24 h significantly reduced ConA-induced IL-2 secretion in a concentration-dependent manner.

## Discussion

In this project, the bioactive polypeptide encoded by gene *ktx*-*sp2* from Scorpiops Pococki is prepared by genetic engineering and its chromatographic pure peptide is successfully obtained, which effectively overcomes the source limitation of toxin polypeptide that cannot be extracted directly from Scorpiops Pococki which has not been domesticated. According to the results of patch clamp recording, Ktx-Sp2 exhibits different blocking activities on three types of voltage-gated potassium channels (Kv1.1, Kv1.2 and Kv1.3) expressed in HEK293T cell membrane. Among them, the blocking activity on Kv1.3 channel is the strongest, which is 33 times and 4 times of Kv1.1 and Kv1.2, respectively. This suggests that it is necessary to further study the structure–function relationship of Ktx-Sp2 so as to further improve its selectivity for Kv1.3 channel. Sequence analysis showed that Ktx-Sp2 is likely belong to α-KTx 12.5 like scorpion toxin LmKTx10 [7] and II10.5 [6]. It is suggested that these three peptides have a relatively consistent spatial configuration, each containing an α helix and two β sheet chains. The corresponding residues in function dyad of Ktx-Sp2 consists of lysine at position 27 and tyrosine at position 36. Besides the pore blocking part of α-potassium channel toxin, the key domain in the Kv1 channel for the interaction with α-potassium toxin locates in α subunit [16]. The scorpion toxin Ktx-Sp2 has relatively consistent function dyad structure with that of LmKTx10 and II10.5. The difference in their activity is mainly reflected in their molecular affinity with amino acid residues in the turret region of the potassium channel, including strong ionic bond interactions between acid–base amino acids and strong hydrogen bond interactions.

Further sequence alignment analysis shows that the amino acid residues in α helix area of Ktx-Sp2, LmKTx10 and II10.5 are S^11^N^12^Q^11^, S^11^S^12^S^13^ and P^10^Q^11^Q^12^, respectively, which may be one of the key factors leading to the difference in activity of these three peptides on Kv1.3 channel. By analyzing the β sheet region of potassium channel toxin, where function dyad structure is the core, it is found that the similarity of amino acid residues of Ktx-Sp2 and LmKTx10 was extremely high, which may be one of the main reasons why the two have similar blocking activity on Kv1.3 channel. Electrophysiological results showed that the blocking activity of Ktx-Sp2 on Kv1.2 channel is about 57 nM, which is about 10 times higher than on Kv1.1 channel. Sequence analysis of the S5–S6 transmembrane domains of Kv1.1 and Kv1.2 channels and related studies have reported that this may be related to the two key amino acid residues: arginine at 34 and tyrosine at 36 in the β sheet region where Ktx-Sp2 function dyad is located. The hydrophobic interaction between the Y36 residue of Ktx-Sp2 and the V379 residue in the filter region of Kv1.2 channel is very important [17], which provides an important theoretical reference for in-depth research on the selective regulation mechanism of Ktx-Sp2 on Kv1 channel.

In this study, the blocking activity of Ktx-Sp2 on Kv1.3 channel on the T cell membrane is further studied by using Jurkat T cell as the cell model. The results showed that the blocking activity of Ktx-Sp2 on the Kv1.3 channel on the T cell membrane is equivalent to the exogenous expression Kv1.3 on the HEK293T cell membrane. During the activation of Jurkat T cells, the expression level of Kv1.3 channel on the cell membrane increases sharply, and then the opening of K^+^ channel leads to a large outflow of K^+^, resulting in the potential hyperpolarization of the cell membrane to provide electrodynamic force, increasing the free Ca^2+^ concentrations in the cell, and promoting T cells to release large amount of inflammatory cytokine IL-2 [18, 19]. Ktx-Sp2 strongly blocks the Kv1.3 channel in Jurkat T cells, which suggest it may block calcium influx in Jurkat T cells and inhibit the release of inflammatory cytokine IL-2. The results of calcium imaging and immunology experiments in this study provide a positive answer. Ktx-sp2 can effectively reduce the concentration of free Ca^2+^ in Jurkat T cells, thereby inhibiting the activation effect of ConA on Jurkat T cells and significantly reducing the release of inflammatory cytokine IL-2. These results prove that Ktx-Sp2 has a strong inhibitory effect on the autoimmune response.

During the activation of T cells in vivo, the expression of Kv1.3 and KCa3.1 potassium channels on the cell membrane both increased to different degrees [20]. The opening of the two K^+^ channels leads to a large outflow of K^+^, which causes the hyperpolarization of cell membrane potential and promotes the influx of Ca^2+^ through CRAC, inducing a large number of T cells to express and release a variety of inflammatory cytokines [21]. Numerous studies have shown that scorpion toxin peptides acting on Kv1.2 channel also exhibit high blocking activity against KCa3.1 [7, 22]. Interestingly, Ktx-Sp2 shows high blocking activity against both Kv1.2 and Kv1.3 channels, suggesting that Ktx-Sp2 may inhibit the autoimmune response by blocking both Kv1.3 and KCa3.1 channels. The results of this study undoubtedly broaden the range of bioactive peptides for the treatment of autoimmune diseases.

## Data Availability

Not applicable.
